# H_2_O_2_ dynamics in the malaria parasite *Plasmodium falciparum*

**DOI:** 10.1371/journal.pone.0174837

**Published:** 2017-04-03

**Authors:** Mahsa Rahbari, Stefan Rahlfs, Esther Jortzik, Ivan Bogeski, Katja Becker

**Affiliations:** 1 Biochemistry and Molecular Biology, Interdisciplinary Research Center, Justus Liebig University Giessen, Giessen, Hessen, Germany; 2 Molecular Physiology, Institute of Cardiovascular Physiology, University Medical Center, Georg August University Göttingen, Göttingen, Niedersachsen, Germany; Institut national de la santé et de la recherche médicale - Institut Cochin, FRANCE

## Abstract

Hydrogen peroxide is an important antimicrobial agent but is also crucially involved in redox signaling and pathogen-host cell interactions. As a basis for systematically investigating intracellular H_2_O_2_ dynamics and regulation in living malaria parasites, we established the genetically encoded fluorescent H_2_O_2_ sensors roGFP2-Orp1 and HyPer-3 in *Plasmodium falciparum*. Both ratiometric redox probes as well as the pH control SypHer were expressed in the cytosol of blood-stage parasites. Both redox sensors showed reproducible sensitivity towards H_2_O_2_ in the lower micromolar range *in vitro* and in the parasites. Due to the pH sensitivity of HyPer-3, we used parasites expressing roGFP2-Orp1 for evaluation of short-, medium-, and long-term effects of antimalarial drugs on H_2_O_2_ levels and detoxification in *Plasmodium*. None of the quinolines or artemisinins tested had detectable direct effects on the H_2_O_2_ homeostasis at pharmacologically relevant concentrations. However, pre-treatment of the cells with antimalarial drugs or heat shock led to a higher tolerance towards exogenous H_2_O_2_. The systematic evaluation and comparison of the two genetically encoded cytosolic H_2_O_2_ probes in malaria parasites provides a basis for studying parasite-host cell interactions or drug effects with spatio-temporal resolution while preserving cell integrity.

## Introduction

Malaria, caused by the apicomplexan parasite *Plasmodium falciparum* (*P*. *falciparum*), is still one of the world’s most severe human infectious diseases. In 104 countries, mostly in the tropics and subtropics, malaria is presently endemic. In 2015, there were an estimated 438,000 deaths, and 198 million people suffered from malaria worldwide [1]. Reactive oxygen species (ROS) are highly reactive and damaging towards DNA, lipids, and proteins [2]. Hydrogen peroxide (H_2_O_2_) is one of the most important cellular ROS and has crucial regulatory and signaling functions. Within cells H_2_O_2_ can be produced by the mitochondrial respiratory chain, NADPH oxidases, through the enzymatic detoxification of superoxide radicals by superoxide dismutase, or–in *P*. *falciparum*–during hemoglobin degradation [3]. Furthermore, intraerythrocytic parasite stages are exposed to increased ROS formation through the Fenton reaction [3–6]. Accordingly, Atamna and Ginsburg [7] reported that erythrocytes infected with *P*. *falciparum* produced significantly higher amounts of hydroxyl (OH^•^) radicals and H_2_O_2_ when compared to uninfected erythrocytes. Although the cytotoxic effects of H_2_O_2_ are well known, H_2_O_2_ is increasingly recognized as an important regulator of signal transduction in eukaryotes [8,9]. Hydrogen peroxide can act as a signaling molecule by regulating growth factors and cytokines to control cell division, differentiation, and migration. Moreover, H_2_O_2_ can diffuse through biological membranes and thus act as a long-range and fast acting signaling molecule [9,10]. Furthermore, H_2_O_2_ controls protein functions of redox-sensitive proteins by selectively oxidizing cysteine reactive residues [11,12]. In bacteria and unicellular eukaryotes, the induced expression of detoxifying enzymes in response to H_2_O_2_ plays an important role in cell protection against oxidative damage [13–16]. Induction of antioxidant gene expression in response to non-lethal doses of H_2_O_2_ allows adaptation and survival of yeast cells after subsequent exposure to usually lethal doses of H_2_O_2_ [17–19]. Thus, H_2_O_2_ can be seen as a sensor and alert, preparing the cell to fight against oxidative stress. In *Plasmodium*, a complex network of glutathione- and thioredoxin-dependent reactions contributes to redox homoeostasis [20]. Until now, however, neither molecular targets nor regulatory mechanisms and dynamic changes of H_2_O_2_-mediated signaling in *P*. *falciparum* have been systematically described, although a better understanding of these processes can contribute to deciphering parasite-host cell interactions and mechanisms of drug action and resistance.

As recently reviewed [21], various techniques have been established to determine redox changes in malaria parasites. These include biochemical methods and application of fluorescent dyes. Although these approaches can be very useful in assessing parasite redox status, they have some serious pitfalls and are prone to artifacts [21]. The development of the genetically encoded H_2_O_2_ sensors roGFP2-Orp1 and HyPer paved the way for non-disruptive, ratiometric, real-time, dynamic, specific, and subcellular compartment-specific measurements of changes in H_2_O_2_ concentration within a living cell [11,22–25]. These sensors are highly selective and sensitive since they can detect submicromolar concentrations of H_2_O_2_. RoGFP2-Orp1 consists of a highly sensitive thiol peroxidase (Orp1) fused to a redox-sensitive green fluorescent protein (roGFP2). Exposure to H_2_O_2_ leads to oxidation being passed from H_2_O_2_ via Orp1 to roGFP2, resulting in an almost stoichiometric oxidation of the probe [26]. The excitation maxima of roGFP2 are at 390 nm and 480 nm (emission at 510 nm). When roGFP2 is oxidized, the excitation peak at 390 nm increases, while the 480 nm peak decreases [26]. HyPer consists of a circularly permuted YFP (cpYFP) inserted into the regulatory domain (RD) of the bacterial H_2_O_2_-sensing protein OxyR [22,27]. Here, we used HyPer-3, which had been developed to improve oxidation-reduction characteristics [23]. In the presence of H_2_O_2_, OxyR is oxidized and changes its conformation [22,27,28]. HyPer-3 possesses two excitation peaks at 420 nm and 500 nm and one emission peak at 530 nm. When exposed to H_2_O_2_, HyPer-3 shows a shift in its excitation spectrum; the excitation peak at 420 nm decreases, while the one at 500 nm increases proportionally [22]. To account for HyPer’s pH sensitivity, its redox insensitive but pH sensitive form SypHer has been used as a control to adjust for artifacts via pH changes [29].

Here, we successfully transfected 3D7 *P*. *falciparum* parasites with the probes roGFP2-Orp1, HyPer-3, and SypHer and directly characterized the functionality of the redox and pH sensors *in vitro* and in cell culture. Furthermore, we studied the effects of antimalarial drugs on the cytosolic H_2_O_2_ level in *Plasmodium* using the redox probes in combination with confocal live cell imaging. Based on our data, both roGFP2-Orp1 and HyPer-3 probes are reliable and valuable tools for studying H_2_O_2_ metabolism in living malaria parasites. However, the necessity to use a pH probe in parallel makes utilizing HyPer-3 more challenging and time consuming.

## Results

### Exclusion of direct interaction of antimalarial drugs with recombinant roGFP2-Orp1 and HyPer-3 *in vitro*

As a prerequisite for our studies, we verified that the redox probes roGFP2-Orp1 and HyPer-3 are both reliable tools for measuring H_2_O_2_ concentrations with actually similar dynamic ranges *in vitro* (S1 Fig). In order to differentiate between pharmacologic effects of antimalarial drugs on parasite H_2_O_2_ signaling and the direct drug-probe interactions, we first performed *in vitro* studies with the recombinant proteins roGFP2-Orp1 and HyPer-3. The tested drugs included artemisinin (ART), artemether (ATM), and artesunate (ATS); the quinoline drugs chloroquine (CQ), mefloquine (MQ), and quinine (QN); the redox cycler methylene blue (MB); and the ellagic acid (EA) derivatives flavellagic acid (FEA) and corulleoellagic acid (CEA). All compounds were tested at concentrations of 1 μM to 1 mM in standard reaction buffer. S1 and S2 Tables summarize the effects of the compounds on the fluorescence ratios 390/480 nm and 500/420 nm of recombinant roGFP2-Orp1 and HyPer-3, respectively, as determined in a plate reader after 0 min, 5 min, 4 h, and 24 h incubation at 25°C. As shown, even high drug concentrations of 1 mM ART, ATM, ATS, CQ, MQ, and QN hardly affected the fluorescence ratio of roGFP2-Orp1 (S1 Table) or HyPer-3 (S2 Table). In contrast, the redox cycler MB and the EA derivatives FEA and CEA led to a significant, immediate increase in fluorescence ratios of both probes, an effect that became visible even at the lowest concentration of 1 μM. For concentrations between 50 μM and 1 mM and longer incubation times, overoxidation of the probes indicated by a decrease in the ratios was also observed (data not shown). Therefore, these compounds were excluded from cell culture studies.

### Different susceptibility of SypHer and HyPer-3 to varying pH values *in vitro* and in cells

Due to the response of HyPer-3 to H_2_O_2_ as well as to pH changes, the direct pH control SypHer, which has been reported to have similar pH sensitivity [29,30], was also characterized *in vitro* and in cells. HyPer-3 and SypHer were successfully expressed and located in the cytosol of *P*. *falciparum* (see below), however, only a rather low transfection rate of about 10% of enriched trophozoites was reached for both HyPer-3 and SypHer. The *in vitro* pH-sensing properties of HyPer-3 and SypHer were investigated with the isolated recombinant proteins. A spectral excitation scan (emission 530 nm) of SypHer showed the same excitation maxima as HyPer-3 at 420 nm and 500 nm for the two conformations of the protein (data not shown) as reported before [22,30]. At pH 4.5 to 7.5 the fluorescence signal at 420 nm increased, while an increase in pH (> pH 8) shifted the fluorescence signal towards the 500 nm peak, confirming its ratiometric properties in response to pH. As shown in S2A Fig, both proteins showed an exponential relationship between 500/420 nm ratio and pH in the pH range of 5.5 to 8.5 *in vitro* (20 min incubation). This was in principle also observed via confocal laser scanning microscopy (CLSM) (488/405 ratio) for the probes targeted to the parasite cytosol (S2B Fig). However, both *in vitro* and in cell measurements showed a slightly different susceptibility of the sensors to pH changes as indicated by lower fluorescence ratios for SypHer. Only between pH 7 and 7.5 did 3D7^[SypHer]^ and 3D7^[HyPer-3]^ parasites exhibit the same fluorescence ratio. Therefore, in *P*. *falciparum* SypHer can–under the given conditions–only be recommended as a pH control for HyPer-3 in the pH range of 7.0–7.5. As indicated by *in vitro* and in-cell data, a pH greater than 7.5 seemed to increase the fluorescence ratio of HyPer-3 more than that of SypHer. Furthermore, the fluorescence ratio of SypHer starting from pH 7.0 *in vitro* remained stable over time (S2C Fig), whereas HyPer-3 was found to increase constantly (S2D Fig). Determining the cytosolic pH of *P*. *falciparum* with SypHer and a pH calibration curve revealed a value of 7.17 ± 0.11. An example of a calibration curve is given in S2E Fig.

### The roGFP2-Orp1, HyPer-3, and SypHer sensors were successfully expressed in *P*. *falciparum* 3D7

The redox and pH sensors were cloned under the control of the pfcrt promoter [31]. Parasite appearance in cell culture occurred 3 weeks after episomal transfection. Full-length expression of the probes in transfected *P*. *falciparum* 3D7 trophozoites was analyzed via western blot. The full-length roGFP2-Orp1 (49 kDa), HyPer-3 (52 kDa) and SypHer (52 kDa) probes were clearly detectable on protein level. For roGFP2-Orp1 proteins with a size of approximately 27 kDa (corresponding to roGFP2 without Orp1 and linker sequence) were also observed (S3 Fig), which might represent degraded proteins as artifacts upon cell lysis or not fully transcribed/translated proteins. Due to the ratiometric readout of the probe, absolute levels of intact probe are not considered crucial as long as the fluorescence signal is strong enough to be determined.

### 3D7^[HyPer-3]^ parasites exhibit a higher H_2_O_2_ sensitivity and a faster oxidation-reduction rate than 3D7^[roGFP2-Orp1]^ parasites

**Fig 1:**
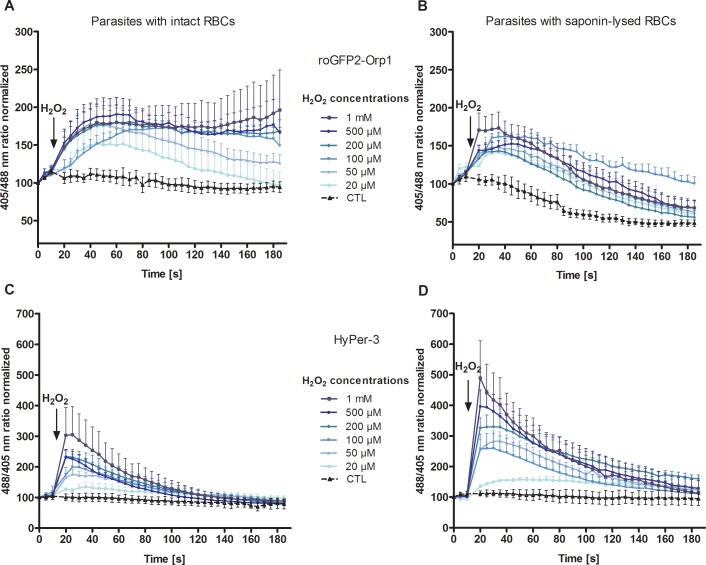
Effect of H_2_O_2_ on *P*. *falciparum* 3D7^[roGFP2-Orp1]^- and 3D7^[HyPer-3]^-transfected parasites within intact RBCs and after RBC lysis_._ After 15 s baseline monitoring, 3D7^[roGFP2-Orp1]^ (**A**) and 3D7^[HyPer-3]^ parasites (**C**) in intact RBCs as well as 3D7^[roGFP2-Orp1]^ (**B**) and 3D7^[HyPer-3]^ parasites (**D**) after saponin lysis of their host cells were exposed to H_2_O_2_ (20 **μ**M to 1 mM) and monitored for 3 min at the CLSM. Non-treated parasites served as controls. In both setups, 3D7^[HyPer-3]^ showed a higher sensitivity to H_2_O_2_ than 3D7^[roGFP2-Orp1]^. Moreover, the H_2_O_2_ response of 3D7^[HyPer-3]^ increased by a factor of 1.6 after lysis of the host cell (**D**). The initial response of 3D7^[roGFP2-Orp1]^ in intact RBCs (**A**) and after saponin-lysis of the host cells (**B**) was comparable; however, in (**B**) the constant decrease of the fluorescence ratio in treated parasites and controls indicates bleaching of the sensor. For each H_2_O_2_ concentration, data from nine trophozoites in total, examined in three independent experiments, were analyzed per data point. Mean and standard error of the mean (SEM) are shown.

The redox probes roGFP2-Orp1 and HyPer-3 were successfully targeted to the cytosol of *P*. *falciparum* 3D7. The expression rate in 3D7^[roGFP2-Orp1]^-enriched trophozoites was around 60%, whereas only 10% of the enriched trophozoites expressed HyPer-3. This allowed for the assessment of H_2_O_2_ susceptibility and dynamic range (see below), however, more complex in-cell studies were not carried out with HyPer-3. In order to measure the effects of H_2_O_2_ on the redox probes in the parasites, enriched 3D7^[roGFP2-Orp1]^ (Fig 1A) and 3D7^[HyPer-3]^ trophozoites (Fig 1C) were exposed to H_2_O_2_ concentrations ranging from 20 μM to 1 mM and monitored for 3 min at the CLSM. For live cell imaging, only parasites that showed fluorescent signals at both 405 and 488 nm excitation and with an intact host cell were chosen. The probes were calibrated with 10 mM DTT for the fully reduced and 1 mM DIA for the fully oxidized state (2 min incubations). To better compare the functionality of the two sensors the obtained ratio values of a time course were all related to the first basal ratio value, which was set to 100. To monitor potential effects of the excitation light on the redox state of the cells, trophozoites were imaged for 3 min under the same experimental conditions but without treatment. Upon adding H_2_O_2_, both 3D7^[roGFP2-Orp1]^ and 3D7^[HyPer-3]^ transfectants showed a concentration-dependent increase in the 405/488 nm and 488/405 nm ratios, respectively (Fig 1A and 1C), in which 3D7^[HyPer-3]^ showed a higher H_2_O_2_ susceptibility and faster oxidation (within seconds) and re-reduction rates than 3D7^[roGFP2-Orp1]^. Oxidation of 3D7^[roGFP2-Orp1]^ at 100 μM to 1 mM H_2_O_2_ remained rather constant over time. As determined by CLSM, both probes were only present in the parasites’ cytosol. However, after short-term treatment with high H_2_O_2_ concentrations (100 μM and higher), a leakage of the roGFP2-Orp1 and HyPer-3 sensors into the erythrocyte cytosol became visible.

In order to determine the influence of the erythrocyte, which surrounds the parasite and might quench H_2_O_2_ before it reaches the redox probes, additional experiments with isolated parasites (directly after saponin lysis of the infected red blood cells (iRBC)) were carried out (Fig 1B and 1D). Treatment of saponin-lysed 3D7^[roGFP2-Orp1]^ iRBCs with H_2_O_2_ again showed time and dose-dependent sensor responses (Fig 1B); however, 1 mM H_2_O_2_ led to a faster oxidation of the probe than in parasites within intact RBCs (Fig 1A). Notably, a strong decrease of the fluorescence ratio occurred over time in controls and treated parasites, which is most likely due to an H_2_O_2_- and light-induced bleaching of the probe (Fig 1B) [32]. In contrast, 3D7^[HyPer-3]^ parasites after RBC lysis exhibited a stable control but in comparison to parasites in intact RBCs (Fig 1C) a more pronounced increase (by a factor of 1.4 to 1.6) in the 488/405 nm ratio upon addition of 50 μM to 1 mM H_2_O_2_ (Fig 1D). Therefore, both experiments (Fig 1B and 1D) indeed indicate that the parasites are more susceptible to H_2_O_2_ after removal of the host cell; however, the two redox probes react differently to the oxidative challenge imposed on host cell-deprived parasites.

### The redox probe HyPer-3 has a higher dynamic range than roGFP2-Orp1 in living parasites

**Fig 2:**
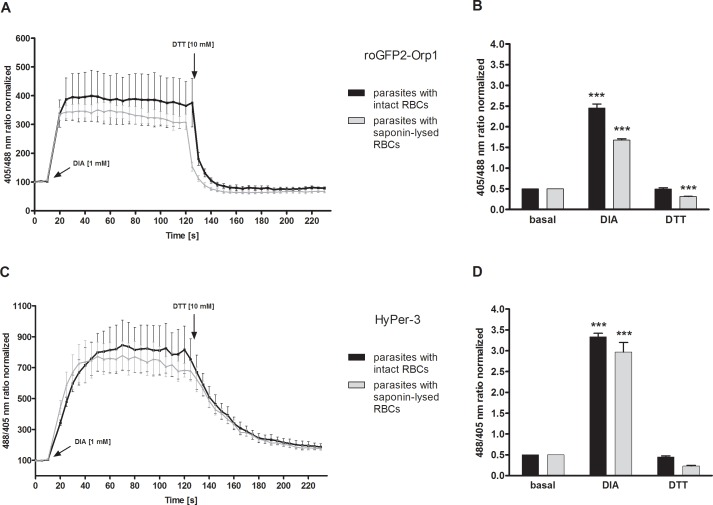
Dynamic range of roGFP2-Orp1 and HyPer-3 in transfected parasites within intact RBCs and after host cell lysis. After 15 s baseline monitoring, 3D7 parasites with intact or lysed host cells and transfected with roGFP2-Orp1 (**A**) or HyPer-3 (**C**) were exposed to 1 mM DIA and monitored for 2 min before adding 10 mM DTT at the CLSM. The fluorescence ratios (405/488 nm, 3D7^[roGFP2-Orp1]^ and 488/405 nm, 3D7^[HyPer-3]^) (**A, C**) at different time points are plotted against time. 3D7^[HyPer-3]^ (**C**) showed a higher DIA sensitivity than 3D7^[roGFP2-Orp1]^ (**A**) in both parasites residing in intact RBCs and those deprived of their host cell. Data from at least three trophozoites in three independent experiments were analyzed per data point. For measuring the dynamic range of both redox sensors in the parasites, the 405/488 nm ratio (3D7^[roGFP2-Orp1]^) (**B**) and the 488/405 nm ratio (3D7^[HyPer-3]^) (**D**) of fully oxidized and reduced probes were computed. The basal ratio, the ratio for 1 mM DIA, and 10 mM DTT after 2 min incubation (n > 27) of 3 independent experiments are shown. 3D7^[roGFP2-Orp1]^ (**B**) and 3D7^[HyPer-3]^ (**D**) with intact RBCs exhibited dynamic ranges of 5 and 7.3, respectively. The dynamic ranges for parasites after RBC lysis of 3D7^[roGFP2-Orp1]^ (**B**) and 3D7^[HyPer-3]^ (**D**) were 5.5 and 12.6, respectively. Mean values and standard error of the mean (SEM) are shown for all experiments. A one-way ANOVA test with 95% confidence intervals with the Dunnett’s Multiple Comparison Test was applied for statistical analysis of significance (*, p < 0.05; **, p < 0.01; ***, p < 0.001).

**Fig 3:**
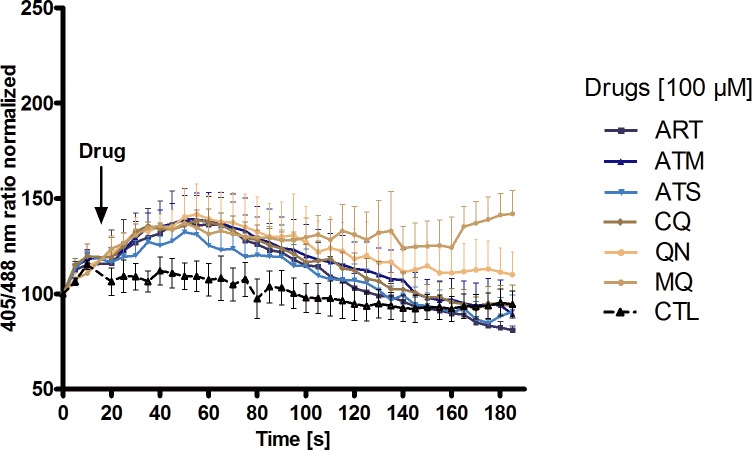
Short-term effects of antimalarial drugs on the redox ratio of *P*. *falciparum* 3D7^[roGFP2-Orp1]^-transfected parasites. After 15 s baseline monitoring, the parasites were exposed to 100 **μ**M antimalarial drugs and monitored for 3 min at the CLSM. Upon addition of all drugs, an increase in the 405/488 nm ratio was detected which was more pronounced for the quinolines, particularly MQ. Each data point (mean ± SEM) is composed of values from nine trophozoites analyzed in three independent experiments.

In order to measure the direct interaction of DIA with roGFP2-Orp1 and HyPer-3 in cells, 3D7^[roGFP2-Orp1]^- and 3D7^[HyPer-3]^-enriched trophozoites (intact RBCs and saponin-lysed RBCs) were exposed to 1 mM DIA and monitored for 2 min at the CLSM. Reduction capacity after DIA treatment was investigated via subsequent treatment with 10 mM DTT. For measuring the presumably completely oxidized and reduced state of the probes, cells were incubated for 2 min with 1 mM DIA and 10 mM DTT, respectively, and blocked thereafter with 2 mM NEM (pretests with NEM are shown in S4 Fig). In order to calculate the dynamic range of the two redox probes in the parasites, the fluorescence ratio of fully oxidized 3D7^[roGFP2-Orp1]^ and 3D7^[HyPer-3]^ was divided by the fluorescence ratio of the fully reduced state. As shown in Fig 2, 1 mM DIA treatment led to a significant increase of the fluorescence ratio of both 3D7^[roGFP2-Orp1]^ (Fig 2A and 2B) and 3D7^[HyPer-3]^ transfectants (Fig 2C and 2D). 3D7^[roGFP2-Orp1]^ within intact RBCs showed a 4.8-fold increase and after RBC lysis a 3.4-fold increase in the 405/488 nm ratio (Fig 2B). 3D7^[HyPer-3]^ within intact RBCs showed a 6.6-fold increase and after RBC lysis a 5.6-fold increase in the 488/405 nm ratio (Fig 2D). Interestingly, roGFP2-Orp1 (Fig 2A) was oxidized more rapidly in the parasites than HyPer-3 (Fig 2C) and recovered quickly after the addition of 10 mM DTT to values below the basal state. The dynamic ranges of 3D7^[roGFP2-Orp1]^ parasites within intact RBCs and parasites after RBC lysis were determined to be 5 and 5.5, respectively. 3D7^[HyPer-3]^ showed a dynamic range of 7.3 within intact RBCs and about 12 after lysis of the host cells (Fig 2B and 2D).

### Effects of artemisinins and quinolines on the H_2_O_2_ levels of roGFP2-Orp1-transfected parasites

In order to test if antimalarial drugs can directly affect the H_2_O_2_ level in *P*. *falciparum*, short-, medium-, and long-term experiments were carried out with 3D7^[roGFP2-Orp1]^-transfected parasites at the CLSM. Studies with HyPer-3 were not further included since its low expression rate in *Plasmodium* and its pH sensitivity required time-consuming SypHer controls. Additionally, preceding experiments had indicated that only between pH 7.0–7.5 can SypHer be used as a pH control (S2B Fig). Fig 3 shows the increase in the 405/488 nm fluorescence ratio of 3D7^[roGFP2-Orp1]^-enriched trophozoites after short-term treatment (3 min) with high concentrations (100 μM) of selected antimalarial drugs. The artemisinins ART, ATM, ATS and the quinolines CQ, QN, and MQ led to an approximately 1.4-fold increase in fluorescence ratio (Fig 3), which resembles an addition of 20 μM H_2_O_2_ (Fig 3). Whereas the increase induced by MQ remained rather constant over the time of the experiment, it was transient for all other drugs. Control studies excluded a direct interaction of the chosen drugs with the purified H_2_O_2_ probe.

In order to investigate mid-term effects of antimalarial drugs and redox-active compounds on roGFP2-Orp1 in living parasites, 3D7^[roGFP2-Orp1]^-enriched trophozoites were exposed to 1 x, 25 x, 50 x, and 100 x EC_50_ of ART, ATM, ATS, QN, CQ, and MQ, incubated for 4 h and subsequently blocked with 2 mM NEM. EC_50_ values determined in the 72 h hypoxanthine incorporation assay were confirmed to be in the known nanomolar range and are shown in S3 Table. For long-term drug exposure experiments (24 h), ring stage parasites were incubated with 4 x EC_50_ of the drugs. Prior to enrichment, cysteines were blocked with 2 mM NEM. None of the tested drugs showed significant effects on the 405/488 nm ratio in either 4 h or 24 h incubations. However, trends towards oxidation were observed for 4 h incubation with ART, ATM (50 x EC_50_, 1.3-fold, respectively), MQ (100 x EC_50_, 1.5-fold), and for 24 h incubation with ART (1.2-fold) (data not shown).

### Priming *P*. *falciparum* 3D7^[roGFP2-Orp1]^-transfected parasites with antimalarial drugs and heat shock affects their H_2_O_2_ susceptibility

**Fig 4:**
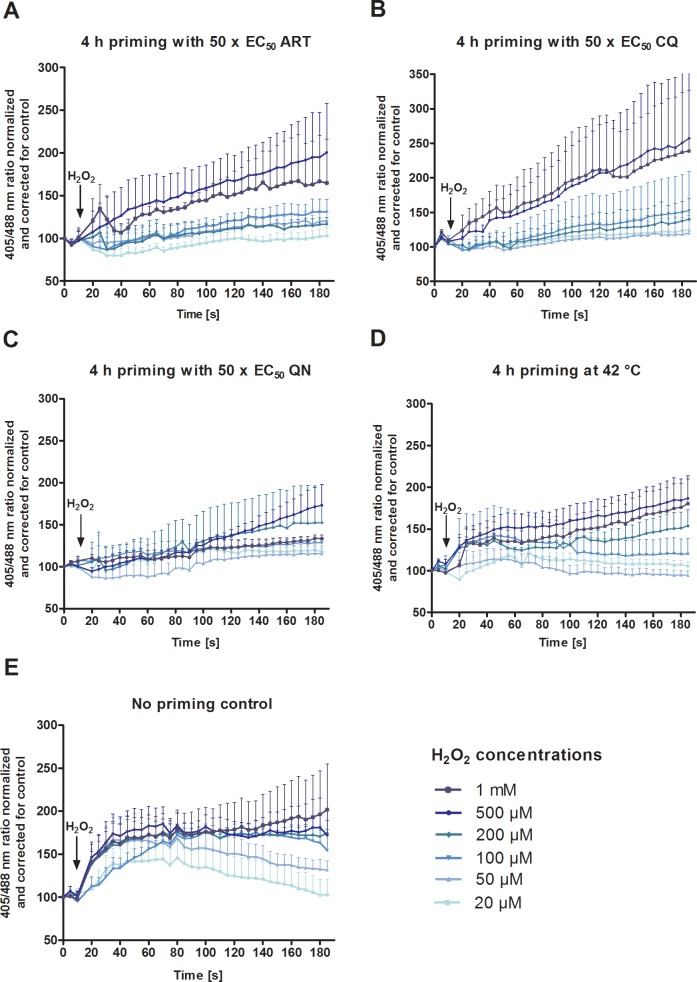
Real-time imaging of the effect of H_2_O_2_ on *P*. *falciparum* 3D7^[roGFP2-Orp1]^-transfected parasites after 4 h pre-incubation with antimalarial compounds and heat shock. 3D7^[roGFP2-Orp1]^-parasites were exposed for 4 h to 50 x EC_50_ ART (**A**), CQ (**B**), QN (**C**), or 42°C (**D**). After 15 s of basal ratio measurement, 20 **μ**M to 1 mM H_2_O_2_ was added and fluorescence was monitored over 3 min at the CLSM. The increases in 3D7^[roGFP2-Orp1]^ fluorescence ratios (405/488 nm) at different time points are plotted against time. Additionally, this ratio increase was corrected for the control in all experiments, and the control without stressors (**D**) served as a reference experiment. Each data point (mean ± SEM) is composed of values from nine trophozoites analyzed in three independent experiments.

Parasites were incubated for 4 h with 50 x EC_50_ ART, CQ, or QN, or at 42°C (Fig 4) and then further challenged with different concentrations of H_2_O_2_ in a time course of 3 min at the CLSM. Additionally, the increase in fluorescence ratio was corrected for the control (without H_2_O_2_), and the non-priming control served as a reference experiment. Interestingly, pre-incubation of 3D7^[roGFP2-Orp1]^-enriched trophozoites with ART, CQ, or QN led to a slower initial increase in the redox ratio upon H_2_O_2_ when compared to the control. This effect was most pronounced in QN pretreated parasites. After a few seconds, however, a constantly progressing oxidation of the probe was observed, which was particularly strong in the CQ pretreated parasites and at higher H_2_O_2_ concentrations (Fig 4A, 4B and 4C). In contrast, 20 μM to 200 μM H_2_O_2_ hardly affected the 405/488 nm ratio of the sensor in pretreated parasites, where only a slight but constant oxidation of the probe could be observed over time. A higher redox stress tolerance was also observed in parasites pre-incubated for 4 h at 42°C (Fig 4D).

## Discussion

### roGFP2-Orp1 and HyPer-3 as dynamic biosensors in *P*. *falciparum*

H_2_O_2_ plays a crucial role in cellular redox signaling. In order to establish a tool for studying H_2_O_2_ fluctuations in intact malaria parasites within one of their native environments, we characterized the redox probes roGFP2-Orp1 and HyPer-3 *in vitro* and in cells. Live cell imaging with the episomally expressed sensors showed that they can be employed as reliable tools with individual strengths.

In line with previous studies in cell systems other than *Plasmodium* [23,26], we determined a rapid, dynamic, and ratiometric response of roGFP2-Orp1 and HyPer-3 *in vitro* upon oxidation with DIA or H_2_O_2_ and reduction with DTT (S1 Fig). The highly efficient thiol-disulfide exchange in roGFP2-Orp1 [26] was confirmed via full oxidation of the probe at equimolar concentrations of H_2_O_2_ (4.7-fold ratio increase). To fully oxidize HyPer-1, Belousov et al. [22] applied a 10-fold molar excess of H_2_O_2_, resulting in a 3.3-fold change in excitation ratio, which was comparable to our results (3.8-fold increase). In our experiments, both probes were suitable for monitoring low H_2_O_2_ *in vitro* and in cells; however, HyPer-3 seemed better suited for monitoring higher H_2_O_2_ concentrations in the parasites.

The dynamic range of roGFP2-Orp1 (DIA/DTT) was determined to be 7.2 *in vitro*, which is comparable to a value of 8 reported by Gutscher et al. [26], but it was only 5 in cells. A dynamic range of approximately 6 has been reported in *Drosophila* [33]. The susceptibility to low or high molecular weight antioxidants might be the reason for the *in vitro*/in-cell differences. Interestingly, for HyPer-3 the *in vitro* dynamic range (5.0) was comparable to the in-cell value (5.5). In zebrafish a dynamic range of only 2.5 was reported for HyPer-3 [23]. On the other hand, 100 **μ**M H_2_O_2_ induced a roGFP2-Orp1 dynamic range of 4.8 in HeLa cells [26], which is higher than the range of 1.7 found in our study after expression in *Plasmodium*. This might be explained by a more efficient H_2_O_2_ detoxification in *Plasmodium* than in HeLa cells. After adding saturating concentrations of 100 **μ**M H_2_O_2_ to HeLa cells expressing HyPer-2, the 500/420 nm ratio of the probe changed 6- to 7-fold [34], which is again higher than the value of 2 for iRBCs and 2.6 for isolated parasites observed with transfected HyPer-3 in our study. The minimal response of a new version of HyPer (HyPerRed) was detected at 10 **μ**M H_2_O_2_ in *E*. *coli* cells [35], which is close to the concentration required to activate HyPer and wild type OxyR in cells [23,29,36]. Similar observations were made at 5–25 **μ**M for HyPer in *E*. *coli* or eukaryotic cytoplasm [22]. Contrary to the *in vitro* results in our study, Gutscher et al. [26] found a similar response of HyPer-1 and roGFP2-Orp1 to H_2_O_2_ expressed in HeLa cells, concluding that the probes have a comparable sensitivity in cells. Moreover, HyPer-1 responded more quickly in comparison to roGFP2-Orp1 in cells, which we have also observed. The authors attribute the slower response of roGFP2-Orp1 to the decelerated reaction between the thiol-disulfide exchange of two protein domains compared to one disulfide bridge formation within HyPer-1 [26]. The difference in the minimal amount of H_2_O_2_ required to fully oxidize HyPer-3 and roGFP2-Orp1 *in vitro* (20 **μ**M and 5 **μ**M, respectively) and in living malaria parasites (1 mM) is likely due to the antioxidant capacity of the parasite-host cell unit including catalase and peroxiredoxins [37]. Notably, HyPer-3 has a faster and higher oxidation-reduction rate towards H_2_O_2_ than roGFP2-Orp1, when expressed in *Plasmodium* (Fig 1). The thioredoxin system (TrxS) (consisting of Trx1, Trx reductase, and NADPH) is the natural reductant of the Orp1 intramolecular disulfide. The stoichiometric response to 1 **μ**M H_2_O_2_ can be fully neutralized in the presence of a TrxS, and a 10-fold molar excess of H_2_O_2_ (10 **μ**M) was mostly neutralized [26]. The oxidation of both probes is reversible, which was demonstrated by adding DTT that decreased the ratio to the baseline redox state by reducing the disulfide bonds (Fig 2). This reversibility also shows the suitability of both sensors to measure dynamic redox changes in living cells, which is an advantage over other currently available imaging agents such as Amplex Red [38]. RoGFP2-Orp1 could be reduced with 10 mM DTT within 5 min, whereas 20 mM DTT and 15 min were needed for HyPer-3 to reach the baseline ratio level (S1 Fig). Notably, the *in vitro* measurements showed a proceeding oxidation of HyPer-3 by air over time (S1D Fig), as previously seen for roGFP2 by Dooley et al. [39]. Therefore, inclusion of controls and/or measurements under anaerobic conditions should be considered for further *in vitro* experiments. Under cell culture conditions, this is less problematic since the sensors seemed not to be affected by air due to the well-buffered cellular environment. Even high percentages of oxygen (100%) could not oxidize roGFP2 in the cytoplasm in different cell types [39].

To investigate to which extent the erythrocytes quench H_2_O_2_ before it reaches the parasite, experiments after RBC lysis were performed. In comparison to roGFP2-Orp1, HyPer-3 exhibited a higher dynamic range in cells for both parasites with (7.3) and without (12.6) intact RBCs, which might imply a higher in cell sensitivity of HyPer-3 to oxidation (Fig 1) and/or a higher reduction of roGFP2-Orp1 by the efficient TrxS in *Plasmodium*. The TrxS had already been identified to enzymatically reduce OxyR under oxidative stress [40], although glutaredoxin 1 seems to be the preferred reductant of OxyR in cells [36].

### The use of SypHer as pH control

Due to the pH sensitivity of HyPer-3, SypHer has been proposed as a pH control [29]. Indeed, our studies also confirmed the results of Poburko et al. [30]: alkaline pH increased and acidic pH decreased the redox ratio of both probes (S2A Fig). Furthermore, SypHer was found to be insensitive to even 10 mM of H_2_O_2_ (data not shown). However, for HyPer-3 we observed a slight but constant increase in fluorescence ratio over time *in vitro* at pH > 7.0, which puts into question the use of SypHer as a direct pH control for certain pH ranges (S2C and S2D Fig). In the next step H_2_O_2_ dose-response curves were generated for both probes in cells, indicating that only in the pH range of 7.0–7.5 were the ratio responses of SypHer and HyPer-3 identical (S2B Fig). We therefore propose that SypHer can be used as a direct pH control for HyPer-3 only in this pH range. We furthermore determined a cytosolic pH of 7.17 ± 0.11 when employing SypHer as a pH probe (S2E Fig). This value is supported by previous studies using a pH-sensitive green fluorescent protein (pHluorin), where a pH of 7.15 was proposed for the cytosol in CQ-resistant and sensitive *P*. *falciparum* parasites [41].

### Direct interactions with the redox probes

Artemisinins and quinolines did not cause an increase in the fluorescence ratio, even at concentrations up to 1 mM and after 24 h incubation, whereas the ellagic derivatives CEA and FEA (polyphenolic lactones), as well as the redox cycler MB, [42] led to direct interaction with both roGFP2-Orp1 and HyPer-3 (S1 and S2 Tables). MB has multifactorial activity comprising enhanced production of H_2_O_2_, loss of NAD(P)H, interference with disulfide reductases, DV basification, and inhibition of heme crystallization [3,42–44]. CEA and FEA lead to improved inhibition of glutathione *S*-transferase, GR, and thioredoxin reductase, as well as heme aggregation due to the higher hydrophilicity compared to EA [45]. However, possible effects of CEA, FEA, and MB on intracellular H_2_O_2_ concentrations cannot be reliably detected by the probes due to their direct interaction with both roGFP2-Orp1 and HyPer-3. It should be noted that in the parasites the observed interactions are likely to be less prominent due to ADME factors resulting most likely in much lower concentrations than those in cells present in the cytosol.

### Effects of antimalarial drugs on cytosolic HyPer-3 and roGFP2-Orp1 in *Plasmodium*

Artemisinin is a traditional Chinese medical herb from *Artemisia annua* with a sesquiterpene lactone endoperoxide structure [46]. The antimalarial effect of ART and its derivatives is mediated by the endoperoxide moiety that interacts with reduced heme generated from hemoglobin degradation. This leads to radical formation that causes damage to parasite cellular macromolecules, including proteins and lipids [47,48]. ART targets DNA synthesis, glycolysis, and hemoglobin digestion pathways within the intraerythrocytic life stage of the parasite [47]. Furthermore, ART has been shown to affect the mitochondrial membrane potential (Δᴪ_m_) [49,50]. Recently, mutations in the propeller domain of the kelch protein K13 of *P*. *falciparum* were revealed to be markers of ART resistance, whereas K13 is putatively linked to oxidative stress as one possible feature of its functional role in parasites [51–56]. Therefore, artemisinin might have several molecular targets in a cell without clear evidence of which mode of action is mainly responsible for its antiparasitic effect. No direct interactions with *P*. *falciparum* peroxiredoxins could be observed so far (unpublished data, Becker lab), which also makes a reaction with roGFP2-Orp1 unlikely. QN is the active ingredient of the *Cinchona* tree bark (South America), while its derivatives CQ and MQ are synthetically produced [44,57,58]. Quinolines are supposed to act as ferriprotoporphyrin (FP) detoxification inhibitors within the digestive vacuole (DV) of the parasite, thus inhibiting hemoglobin degradation. Sequestration into nontoxic crystalline hemozoin is prevented [3,57]. Glutathione metabolism has been associated with CQ resistance [3,59], in which GSH levels were observed to be lower in the cytosol of CQ-resistant parasite lines [60]. GSH forms a complex with heme in the DV of the parasite in order to prevent oxidative damage, whereas the CQ-heme complex is toxic [61]. CQ resistant parasites harbor multiple mutations in the CQ resistance transporter PfCRT, an integral membrane protein in the DV, with these mutations consistently including K76T, irrespective of their geographic origin [62,63]. Likewise, glutathione transport processes seem to play an important role for CQ resistance. Mutant PfCRTs exhibit glutathione transport activity, in which the transport of GSH into the DV is increased in CQ-resistant parasite lines [60].

In short-term incubation, even 100 μM of antimalarial drugs, a rather high concentration, did not show significant effects on roGFP2-Orp1 expressed in *P*. *falciparum* and therefore most likely did not affect H_2_O_2_ levels (Fig 3). Interestingly, quinolines, especially mefloquine, showed a longer-lasting effect on the redox sensor–a phenomenon that might be worth studying in more detail. 4 h and 24 h incubation with antimalarial drugs did not cause a significant increase in the fluorescence ratio of roGFP2-Orp1 either.

In order to investigate whether pre-incubation with antimalarial drugs or heat shock affects the H_2_O_2_ susceptibility of *P*. *falciparum*, 4 h incubations with ART, CQ, QN, or at 42°C and subsequent exposure to H_2_O_2_ were carried out (Fig 4). Interestingly, under all conditions a slower initial increase of the redox ratio was observed after H_2_O_2_ treatment. This effect was most pronounced in QN-pretreated parasites and is likely due to the induction of antioxidant defense mechanisms such as peroxiredoxins [37,64], which reduce hydrogen peroxide rapidly [65–67]. This indicates that for none of the antimalarial drugs tested here did increased H_2_O_2_ levels play a major role in the mode of action in parasites. Neither immediate nor long-term effects on H_2_O_2_ levels were observed in our experimental setup with the redox probe targeted to the cytosol. It should, however, be considered that the tested drugs do not directly act at the cytosol level but mainly at organelle sites such as digestive vacuole, apicoplast, or mitochondrion, which most likely explains our results. The probes used in this study were also targeted to different organelles in the parasite to determine compartment-specific drug effects, but due to low expression rates and weak fluorescence intensities of the targeted sensors, experimental studies could not be carried out. In the future, the sensors should be stably integrated into the genome of *P*. *falciparum* in order to improve their fluorescence intensities and expression rates. In fact, the *P*. *falciparum* NF54-*attB* strain (CQ-sensitive) and the multidrug-resistant line Dd2-*attB* were engineered for the application of genomic integration using the *attB* × *attP*-based methodology [68,69]. It should be noted that, when using a peroxidase-probe, it is not possible to differentiate between a lack of H_2_O_2_ increase and rapid H_2_O_2_ reduction by endogenous peroxiredoxins or other electron donors. Our results show a higher tolerance towards exogenous H_2_O_2_ after priming with drugs pointing towards upregulation of the peroxide defense system. As the same effect was seen upon heat stress, this might represent a rather general stress-induced upregulation of primary defense systems. Kimura et al. [70] reported that in *P*. *falciparum* TPx-1 (thioredoxin peroxidase 1) disruption renders hypersensitivity to ROS [71], RNS [71], and heat stress, indicating that PfTPx-1 had a hypothermal protective function [70].

## Conclusion

Our results suggest that the genetically encoded H_2_O_2_ biosensors roGFP2-Orp1 and HyPer-3 are both sensitive and valuable tools to study H_2_O_2_ homeostasis in the cytosol of *Plasmodium falciparum*. We furthermore showed that the use of the pH sensor SypHer as a direct control for HyPer-3 is limited to pH values between 7.0 and 7.5. Pre-incubation of *Plasmodium* with antimalarial drugs or heat shock and subsequent exposure to H_2_O_2_ suggest that stress in general influences redox metabolism by upregulating the antioxidative defense system. In the future, a stable genomic integration of the sensors is likely to overcome the limitations of transient transfections, allowing more detailed in-cell studies with the probes. These studies employing the *attB* × *attP*-based methodology are presently conducted in our laboratory.

## Materials and methods

### Drugs and chemicals

All chemicals used were of the highest available purity and were obtained from Roth (Karlsruhe, Germany), Sigma-Aldrich (Steinheim, Germany), or Merck (Darmstadt, Germany). RPMI 1640 medium was obtained from Gibco (Paisley, United Kingdom). ART and MB were from Roth, CQ and ATS from Sigma-Aldrich, MQ from Roche (Mannheim, Germany), and ATM from TCI Germany (Eschborn). CEA and FEA were kindly provided by Herbert Zimmermann (Heidelberg, Germany), and QN was from Acros Organics (Geel, Belgium). WR99210 was kindly supplied by Jacobus Pharmaceuticals, New Jersey, USA. Stock solutions of diamide (DIA), DTT, CQ, and MB were dissolved in sterile distilled H_2_O, while ART, ATM, ATS, MQ, QN, CEA, and FEA were dissolved in DMSO.

### Cloning the roGFP2-Orp1, HyPer-3, and SypHer constructs and heterologous overexpression of the recombinant proteins

Tobias Dick, Heidelberg, kindly provided the sensor roGFP2-Orp1 in pQE60, and Vsevolod Belousov, Moscow, provided HyPer-3 and SypHer in the pHyPer-cyto and the pSypHer-cyto vectors, respectively. To evaluate the *in vitro* interactions of drugs and redox-active compounds with the redox probes roGFP2-Orp1 and HyPer-3, as well as the pH sensor SypHer, we recombinantly produced the respective proteins. For experiments in intact cells, roGFP2-Orp1, HyPer-3, and SypHer were cloned into the pARL1a+ expression vector with the pfcrt 5`promoter [31] using *Kpn*I restriction sites. For heterologous overexpression, HyPer-3 and SypHer were cloned into the pET28a+ expression vector with *Nco*I and *Xho*I restriction sites. All primers used are listed in the supporting information section (S4 Table).

### Heterologous overexpression of recombinant roGFP2-Orp1, HyPer-3, and SypHer

*E*. *coli* M15[pREP4] cells (Kan^R^) were transformed with roGFP2-Orp1 in pQE60 (carbenicillin resistance, Cn^R^) and *E*. *coli* KRX cells were transformed with either HyPer-3 or SypHer in pET28a+ (kanamycin resistance, Kan^R^). A pre-culture in LB medium (containing 100 μg/ml Cn and/or 50 μg/ml Kan) was inoculated with a colony and grown for 6–7 h at 37°C with vigorous shaking. After incubation, the pre-culture was poured into 50 ml LB medium with antibiotics and grown overnight at 37°C with constant shaking. 500 ml of LB medium containing antibiotics was inoculated with 10–15 ml of the overnight culture and grown at 37°C up to an optical density at 600 nm (OD_600_) of 0.1. The culture was induced at an OD_600_ of 0.6 with either 1 mM IPTG (for expression of roGFP2-Orp1) or 0.1% rhamnose (for expression of HyPer-3 and SypHer) and incubated overnight at RT. The cells were harvested via centrifugation (8,000 rpm, 15 min, 4°C), resuspended (1 g pellet/4 ml buffer) in US buffer (50 mM sodium phosphate buffer, 300 mM NaCl, pH 8.0), and mixed with protease inhibitors (150 nM pepstatin, 40 nM cystatin, 100 μM PMSF) before storage at -20°C. The roGFP2-Orp1, HyPer-3, and SypHer proteins were purified via hexahistidyl affinity chromatography on Ni-NTA material, concentrated using 30 kDa Vivaspin columns (Sartorius, Goettingen), and stored at -20°C with 10% glycerol.

### *In vitro* characterization of recombinant roGFP2-Orp1, HyPer-3, and SypHer

All drugs, DIA, DTT, and H_2_O_2_ were diluted with a standard reaction buffer (100 mM potassium phosphate, 1 mM EDTA, pH 7.0) and used immediately. Before the experiments, the reaction buffer was degassed for 30 min at RT. Purified recombinant roGFP2-Orp1 and HyPer-3/SypHer proteins were reduced with 5 mM DTT for 10 min and 20 mM DTT for 30 min, respectively, at 4°C, desalinated (Zeba^TM^ Spin Desalting Columns, Thermo Scientific), and diluted in reaction buffer to a final concentration of 5 **μ**M. A 5-fold drug/redox-active compound dilution (25 **μ**l) was mixed with 100 **μ**l of 5 **μ**M roGFP2-Orp1/HyPer in a 96-well microplate (black, half-area, Greiner Bio-One, Frickenhausen). Prior to fluorescence measurements via plate reader, protein concentration and loading time were optimized. For pH measurements with recombinant SypHer and HyPer-3, pH values of the standard potassium buffer were varied by adding 2[*N*-morpholino]ethanesulfonic acid (MES) at 20 mM and potassium hydroxide (KOH) to 100 ml buffer to lower the pH value. To raise the pH value, tris(hydroxymethyl)aminomethane (Tris) at 20 mM and hydrochloric acid (HCl) were added to 100 ml buffer. The emission of roGFP2-Orp1 after excitation at 390 nm and 480 nm was measured in a plate reader (Infinite M200, Tecan) with optimal read settings. The ratio of the fluorescence signals at 390/480 nm were calculated for roGFP2-Orp1, whereas the ratios at 500/420 nm were calculated for HyPer-3/SypHer and plotted against time or concentration of antimalarial drugs/redox active compounds. For excitation spectrum scans, emission was measured at 510 nm for roGFP2-Orp1 and at 530 nm for HyPer-3 after excitation from 340 to 510 nm and 340 nm to 530 nm, respectively. For time courses, data from three independent experiments were analyzed per data point for each concentration. Mean and standard error of the mean (SEM) are shown.

### Cell culture and transfection of *P*. *falciparum*

The chloroquine (CQ)-sensitive 3D7 strain of *P*. *falciparum* was cultured as described [72]. The strain was propagated in RBCs (A+) in RPMI 1640 medium supplemented with 0.5% Albumax, 9 mM glucose, 0.2 mM hypoxanthine, 2.1 mM L-glutamine, 25 mM Hepes, and 22 μg/ml gentamycin at 3.3% hematocrit (15 ml culture) and 37°C in a gaseous mixture consisting of 3% O_2_, 3% CO_2_, and 94% N_2_. Synchronization of *P*. *falciparum* parasites was carried out with 5% (w/v) sorbitol [73]. *P*. *falciparum* trophozoites were enriched via magnet separation [74]. Cell lysate was obtained via saponin lysis [75]. Parasitemia was counted by using Giemsa-stained blood smears (red blood cell concentrates were purchased from the Blood Bank of the Universitätsklinikum Giessen Marburg (UKGM)).

### Transfection of *P*. *falciparum*

Transfection of *P*. *falciparum* was carried out as described [31]. A 5 ml culture (ring stage 6–10 h, 5–8% parasitemia, 5% hematocrit) was centrifuged (2,100 rpm, 4 min), and the supernatant was aspirated. The parasite pellet (200 μl) was mixed with 150 μg of purified plasmid (Maxi prep Kit) in 400 μL of cytomix (120 mM KCl, 0.15 mM CaCl_2_, 2 mM EGTA, 5 mM MgCl_2_, 10 mM K_2_HPO_4_/KH_2_PO_4_, 25 mM Hepes, pH 7.6) and then electroporated in a 2 mm electroporation cuvette (310 V, 950 μF, capacitance ∞) with the Bio-Rad Gene Pulser [31]. The resulting time constant was between 10 and 15 sec. The electroporated sample was returned to a 15 ml culture with 3.3% final hematocrit. To select for transfectants, 2 nM WR99210 was added to the culture of roGFP2-Orp1, HyPer-3, and SypHer 24 h post transfection. The culture medium of 3D7^[roGFP2-Orp1]^ was changed every day for the first 5 days under constant drug pressure and after that every other day. For 3D7^[HyPer-3]^ and 3D7^[SypHer]^, 2 nM WR99210 was added constantly for 5 days and after that during every second media change. The drug concentration was increased to 5 nM after the appearance of transfectants (usually after 3–4 weeks). After one week, 50 μl fresh RBCs were added to the parasites, and thereafter the culture was cut 1:2 every week until the growth of the parasites.

### Western blot of the roGFP2-Orp1, HyPer-3, and SypHer probes

For western blot analysis, 10 ml of transfected *P*. *falciparum* 3D7 trophozoite stage parasites (30–34 h) (6–8% parasitemia, 5% hematocrit) were harvested via saponin lysis [75,76]. Parasite cultures were centrifuged, the pellets resuspended with 20 volumes of saponin lysis buffer (0.02% saponin, 10 mM NaH_2_PO_4_, 10 mM Na_2_HPO_4_, 145 mM NaCl, 3 mM KCl, pH 7.2), incubated two times for 10 min at 37°C with inverting, and washed three times with phosphate-buffered saline (PBS) at decreasing temperature. Pellets were kept at -80°C until further treatment. For preparing the parasite cell extract, pellets were diluted in an equal volume of PBS and complete protease inhibitor cocktail (Roche, Mannheim, Germany). Parasites were disrupted via four cycles of freezing in liquid nitrogen and thawing in a water bath at RT. After centrifugation at 50,000 rpm for 30 min at 4°C, the obtained supernatant was used for western blotting. 0.2–18 μg of recombinant proteins and proteins from the parasite lysate were separated onto 12% SDS gels and transferred to a PVDF membrane (Roth, Karlsruhe, Germany). Membranes were probed with α-GFP (1:1000; Roche) identification and were followed by secondary antibody α-mouse (1:2000; Dianova, Hamburg, Germany). All antibodies for western blotting were diluted in 5% non-fat milk in TBST.

### Confocal live cell imaging and image processing

Magnetically enriched *P*. *falciparum*-infected erythrocytes (trophozoite stage 26–30 h post invasion) were washed with pre-warmed (37°C) Ringer’s solution (122.5 mM NaCl, 5.4 mM KCl, 1.2 mM CaCl_2_, 0.8 mM MgCl_2_, 11 mM D-glucose, 25 mM Hepes, 1 mM NaH_2_PO_4_, pH 7.4) and prepared as described. 50 μL of cells (1.0 x 10^6^ cells/50 μl) were seeded either onto poly-L-lysine-coated μ-Slides VI for time course experiments or onto poly-L-lysine-coated μ-Slides 18 well (flat) (Ibidi, Martinsried, Germany) for endpoint measurements. A Leica confocal system TCS SP5 inverted microscope equipped with the objective HCX PL APO 63.0x1.30 GLYC 37°C UV connected to a 37°C temperature chamber was used. The argon laser power was set to 20%; scanning was performed at 400 Hz frequency and at a 512×512 pixel resolution. The smart gain and smart offset were 950 V and -0.9%, respectively. With a sequential scan, we excited the probes at 405 nm and at 488 nm and detected emissions at 500–550 nm. Laser intensity for both lines was adjusted to match the full dynamic range of the probes to the dynamic range of the detector (roGFP2-Orp1: 405 nm: 12%, 488 nm: 4%; HyPer-3/SypHer: 405 nm: 15%, 488 nm: 4%). For time series, images were acquired every 5 s over a time course of 3 min after 15 s of basal measurements. Autofluorescence images were simultaneously taken at ex 405 nm / em 430–450 nm and individually defined together with the background for every image, but no fluorescence signal could be detected. The Leica LAS AF Lite software for fluorescence analysis was used. The 405/488 nm or 488/405 nm ratios were calculated. The graphs were plotted using the GraphPad Prism 5 software (San Diego, CA, USA). For analyzing microscope images, ImageJ software was used. For live cell imaging, only parasites showing fluorescent signals at both 405 and 488 nm excitation and an intact host cell were chosen. The laser settings were calibrated with 10 mM DTT for the fully reduced and 1 mM DIA (2 min incubation) for the fully oxidized state. Due to the fluctuations of the basal 405/488 nm and 488/405 nm ratio of single parasites, the obtained ratio values of a time course were all related to the first basal ratio value, which was set to 100. For endpoint experiments the obtained ratio values were normalized to the control value, which was set to 100. To measure potential effects of the lasers on the redox state of the cells, trophozoites were monitored over 3 min without treatment under the same experimental conditions and served as a control. Each incubation time and drug concentration treatment was carried out three times. For each time course, at least three parasites were assessed resulting in at least nine experimental values per data point. Endpoint experiments comprise at least 10 parasites, resulting in at least 30 experimental values per incubation.

### Effects of H_2_O_2_ on the redox homeostasis of *P*. *falciparum* parasites with intact and saponin-lysed RBCs

H_2_O_2_ concentrations of 20 **μ**M, 50 **μ**M, 100 **μ**M, 200 **μ**M, 500 **μ**M, and 1 mM were added to 3D7^[roGFP2-Orp1]^- and 3D7^[HyPer-3]^-transfected parasites in short-term (3 min) time course experiments. In the experiment comprising intact iRBCs, trophozoite stage parasites (26–30 h) were magnetically enriched (Miltenyi Biotec, Germany), counted by using the improved Neubauer hemocytometer (Brand GmbH, Germany), and returned to cell culture for at least 1 h to recover. The cells were washed one time with pre-warmed Ringer’s solution and resuspended in Ringer’s solution with a final parasite concentration of 2.0 x 10^4^ trophozoites/**μ**l. For every H_2_O_2_ concentration, 3 parasites from 3 independent experiments were analyzed per data point. For experiments with lysed erythrocytes, saponin lysis was applied. Trophozoite stage parasites (26–30 h) were centrifuged in a 15 ml conical (5 min, 2,100 rpm, RT). 10 ml pre-warmed saponin lysis buffer was added to the cells and incubated for 10 min in a water bath (37°C), inverting the tube now and then. The parasites were again centrifuged, resuspended with 5 ml saponin lysis buffer, and incubated for 5 min at RT. After another centrifugation step, the cells were washed one time with pre-warmed Ringer’s solution and resuspended in Ringer’s solution with a final parasite concentration of 2.0 x 10^4^ trophozoites/**μ**l. Experiments were performed within 2 h after the end of saponin lysis. All experiments included non-treated, fully reduced, and fully oxidized parasites as controls and were carried out within 6 weeks after the appearance of transfectants. For dynamic range time courses with DIA and DTT, data from three independent experiments of at least three parasites each were analyzed per data point. Mean and standard error of the mean (SEM) are shown. A one-way ANOVA test with 95% confidence intervals with the Dunnett’s Multiple Comparison Test (GraphPad Prism 5.0) was applied for statistical analysis of significance (*, p < 0.05; **, p < 0.01; ***, p < 0.001).

### Effects of antimalarial drugs on redox homeostasis

The effects of antimalarial drugs, DIA, and H_2_O_2_ on *P*. *falciparum* were investigated in short-term (3 min), 4 h, and 24 h incubation experiments. The half-maximal effective concentration (EC_50_) of drugs on *P*. *falciparum* 3D7 asexual blood stages was determined with the [^3^H]-incorporation assay [77] (S3 Table). For short-term and 4 h experiments, trophozoite stage parasites (26–30 h) of 3D7^[roGFP2-Orp1]^ (6–8% parasitemia) were magnetically enriched (Miltenyi Biotec, Germany), counted by using the improved Neubauer hemocytometer (Brand GmbH, Germany), and returned to cell culture (at 2.0 x 10^4^ trophozoites/μl) for at least 1 h to recover. 1.0 x 10^6^ cells in 100 μl cell culture medium were placed into LoBind tubes (Eppendorf) for 4 h incubation experiments. The parasites were treated with antimalarial drugs at 1 x, 25 x, 50 x, 100 x EC_50_ and with the redox-active compounds DIA (1 mM) and H_2_O_2_ (1 mM) for 4 h under cell culture conditions. Subsequently, free thiol groups were blocked with 2 mM N-ethylmaleimide (NEM) for 15 min at 37°C. For 24 h experiments, a 7.5 ml culture (5% hematocrit, 6–8% parasitemia) of ring-stage parasites (6–10 h post invasion) was treated with antimalarial drugs at 4 x EC_50_, DIA (100 μM) and H_2_O_2_ (100 μM). Prior to enrichment, cysteines were blocked with 2 mM NEM. For short-term, 4 h and 24 h experiments, cells were washed after incubation, resuspended in Ringer’s solution, and measured in the Leica confocal system TCS SP5 with excitation wavelengths at 405 nm and 488 nm. In order to investigate the short-term effects of antimalarial drugs and DIA on 3D7^[roGFP2-Orp1]^-infected parasites, 50 μl of cells (1.0 x 10^6^ trophozoites) were challenged with 100 μM of each drug and 1 mM of DIA, and the fluorescence signals were monitored in a time course of 3 min. All experiments included non-treated parasites as controls, and both fully reduced and fully oxidized parasites. Each incubation time and drug concentration treatment was carried out three times. At least nine microscopy images were taken. All experiments were carried out within six weeks after the appearance of transfectants.

### Determination of pH susceptibility of SypHer and HyPer-3 in living parasites

Single cell pH measurements of *P*. *falciparum* cytosol were carried out according to Kuhn et al. (2007) [41]. An in cell fluorescence calibration curve was generated by using sodium-free high potassium buffer (160 mM KCl, 1.2 mM CaCl_2_, 0.8 mM MgCl_2_, 11 mM D-glucose, 25 mM Hepes) of varying pH (5.5–8.5). After magnet enrichment of trophozoites, cells were kept under standard culture conditions for at least 1 h and washed in Ringer’s solution, and 1.0 x 10^6^ cells were transferred to the different potassium-rich calibration buffers. Cells were incubated with 4 μM of the ionophore nigericin for 20 min at RT, and 488/405 nm ratios were subsequently measured with the Leica confocal microscope. A trend line was created via exponential regression, and the pH for the respective 488/405 nm ratio of the non-nigericin-treated cells was calculated.

### Determination of H_2_O_2_ susceptibility of *P*. *falciparum* transfected with roGFP2-Orp1 after priming with stress factors

To determine whether pre-incubation with antimalarial drugs and heat shock would increase the H_2_O_2_ susceptibility of *P*. *falciparum*, priming experiments were performed for 4 h. Trophozoite stages (26–30 h) of 3D7^[roGFP2-Orp1]^-transfected parasites (6–8% parasitemia) were magnetically enriched (Miltenyi Biotec, Germany), counted by using the improved Neubauer hemocytometer (Brand GmbH, Germany), and returned to cell culture (at 2.0 x 10^4^ trophozoites/μl) for at least 1 h to recover. 1.0 x 10^6^ cells in 100 μl cell culture medium were placed into LoBind tubes (Eppendorf) and were treated in four separate experiments with either 50 x EC_50_ of ART, CQ, and QN or as an additional stress factor with heat shock at 42°C for 4 h. After incubation, 50 μl of cells (5.0 x 10^5^ trophozoites) were exposed to varying H_2_O_2_ concentrations of 20 μM, 50 μM, 100 μM, 200 μM, 500 μM, and 1 mM, and the fluorescence signals were monitored in a time course of 3 min. All experiments included pre-incubated, non-H_2_O_2_-treated parasites as controls. Each experiment and H_2_O_2_ concentration treatment was carried out three times.

## Supporting information

S1 Fig Excitation spectra and *in vitro* characterization of recombinant roGFP2-Orp1 and HyPer-3 exposed to H_2_O_2_ and redox active agents. 5 **μ**M of roGFP2-Orp1 (**A**) and HyPer-3 (**B**) were exposed to 1 mM DIA, 10 mM DTT, and different H_2_O_2_ concentrations in a microplate reader, which caused concentration-dependent changes in the excitation spectra. The recombinant proteins roGFP2-Orp1 and HyPer-3 have two excitation maxima at 390 nm and 480 nm (emission at 510 nm) (**A**), and 420 nm and 500 nm (emission at 530 nm) (**B**), respectively. Notably, roGFP2-Orp1 was fully oxidized by 5 **μ**M H_2_O_2_ (**A**), whereas 20 **μ**M H_2_O_2_ had to be applied to fully oxidize HyPer-3 (**B**). Furthermore, after 1.5 min baseline monitoring, recombinant roGFP2-Orp1 (**C**) and HyPer-3 (**D**) were treated with different concentrations of H_2_O_2_, 1 mM DIA, or 10 mM DTT and ratio changes were monitored for 15 min. For each concentration, data from three independent experiments were analyzed per data point. Mean and standard error of the mean (SEM) are shown.
(TIF)

S2 Fig PH calibration curve of SypHer and HyPer-3 of *in vitro* and in cell measurements and their ratio over time (*in vitro*). (**A**) Recombinant SypHer and HyPer-3 were suspended in pH buffers ranging from 5.5 to 8.5. Relative fluorescence units (RFUs) were measured at the emission wavelength of 530 nm and the 500/420 nm ratio was recorded. (**B**) In parallel, *P*. *falciparum* 3D7^[SypHer]^-transfected and 3D7^[HyPer-3]^-transfected parasites were studied at different pH values and the 488/405 ratio was recorded. In both *in vitro* (**A**) and in-cell (**B**) experiments, a different pH sensitivity of SypHer and HyPer-3 became evident. Only between pH 7–7.5 was the pH sensitivity of SypHer and HyPer-3 in the parasites comparable (**B**). Panels (**C**) and (**D**) show a time course for both sensors at different given pH values *in vitro*. Data indicate a higher stability of the SypHer probe. (**E**) Via calibration curves and nonlinear regression using the pH sensor SypHer, the cytosolic pH of *P*. *falciparum* 3D7 was determined to be 7.17 ± 0.11. As indicated, an exponential relationship exists between the 488/405 ratio of the sensor and the pH. For each pH value in *in vitro* measurements with the microplate reader, data from three independent experiments were included per data point. In living parasites, each data point represents ≥ 24 trophozoites analyzed in three independent experiments (CLSM detection). Mean and standard error of the mean (SEM) are shown.
(TIF)

S3 Fig Western blots of roGFP2-Orp1, HyPer-3, and SypHer-transfected parasite lysates and the corresponding recombinant purified proteins. 0.2 **μ**g of each recombinant purified protein was loaded, as well as 5 **μ**g of parasite lysate expressing HyPer-3 and roGFP2-Orp1, respectively. For SypHer parasite lysate, 18 **μ**g had to be applied because of its low transfection rate. Full-length roGFP2-Orp1 (49 kDa), HyPer-3 (52 kDa), and SypHer (52 kDa) probes are highlighted. For roGFP2-Orp1, a second band of ~ 27 kDa was also detected.
(TIF)

S4 Fig Real-time imaging of *P*. *falciparum* 3D7^[roGFP2-Orp1]^-transfected control parasites and with 2 mM NEM pre-blocked parasites after exposure to DIA. *P*. *falciparum* 3D7^[roGFP2-Orp1]^ trophozoites were magnetically enriched and maintained at standard cell culture conditions for 2 h to recover. Parasites were then distributed to LoBind tubes (1 x 10^6^ iRBCs/100 μl) and incubated with 100 μM, 500 μM, 1 mM, 2 mM, 5 mM, 10 mM, 15 mM, and 20 mM NEM for 15 min to block the cysteine SH-groups. Subsequently, the parasites were exposed to 1 mM DIA to test blocking of the different NEM concentrations. The 405/488 nm ratio was measured via CLSM. Only the data of the 2 mM NEM incubation are shown, which was the lowest concentration showing full protective effects.
(TIF)

S1 Table Effects of antimalarial drugs on the redox ratio of recombinant roGFP2-Orp1 *in vitro*. (PDF)

S2 Table Effects of antimalarial drugs on the redox ratio of recombinant HyPer-3 *in vitro*. (PDF)

S3 Table EC_50_ values of antimalarial drugs on *P*. *falciparum* 3D7 determined via the [H]^3^-hypoxanthine incorporation assay described in [77]. (PDF)

S4 Table Primer sequences for cloning roGFP2-Orp1, HyPer-3, and SypHer into the expression vector pARL1a+ for in-cell experiments, and for cloning HyPer-3 and SypHer into the expression vector pET28a+ for *in vitro* experiments. (PDF)

## Abbreviations

ART: artemisinin
ATM: artemether
ATS: artesunate
CEA: coruleoellagic acid
CLSM: confocal laser scanning microscopy
Cn: carbenicillin
cpYFP: circularly permuted yellow fluorescent protein
CQ: chloroquine
DHFR: dihydrofolate reductase
DIA: diamide/diazenedicarboxamide
DMSO: dimethylsulfoxide
DTT: 1,4-dithiothreitol
DV: digestive vacuole
EC_50_: half maximal effective concentration
EDTA: ethylenediaminetetraacetic acid
EA: ellagic acid
FEA: flavoellagic acid
GFP: green fluorescent protein
GR: glutathione reductase
GSH: reduced glutathione
IPTG: isopropyl-β-D-thiogalactopyranoside
iRBC: infected red blood cell
Kan: kanamycin
LB: Luria-Bertani medium
MB: methylene blue
MQ: mefloquine
MES: 2-(*N*-morpholino)ethanesulfonic acid
NADPH: nicotinamide adenine dinucleotide phosphate
NEM: N-ethylmaleimide
OD_600_: optical density at 600 nm
Orp: oxidant receptor peroxidase
PBS: phosphate buffered saline
PCR: polymerase chain reaction
P. falciparum: Plasmodium falciparum
PMSF: phenylmethylsulfonyl fluoride
PVDF: polyvinylidene difluoride
QN: quinine
RBC: red blood cell
RD: regulatory domain
RFU: relative fluorescence units
roGFP: reduction-oxidation sensitive green fluorescent protein
ROS: reactive oxygen species
rpm: revolutions per minute
RT: room temperature
*S. cerevisiae*: *Saccharomyces cerevisiae*
SDS: sodium dodecyl sulfate
SDS-PAGE: sodium dodecyl sulfate polyacrylamide gel electrophoresis
TBST: tris-buffered saline with tween 20
YFP: yellow fluorescent protein
