# Supplementary figures and images for: H2O2 dynamics in the malaria parasite Plasmodium falciparum
Source: PLoS One. 2017 Apr 3;12(4):e0174837. doi: 10.1371/journal.pone.0174837 (PMC5378400; doi:10.1371/journal.pone.0174837)

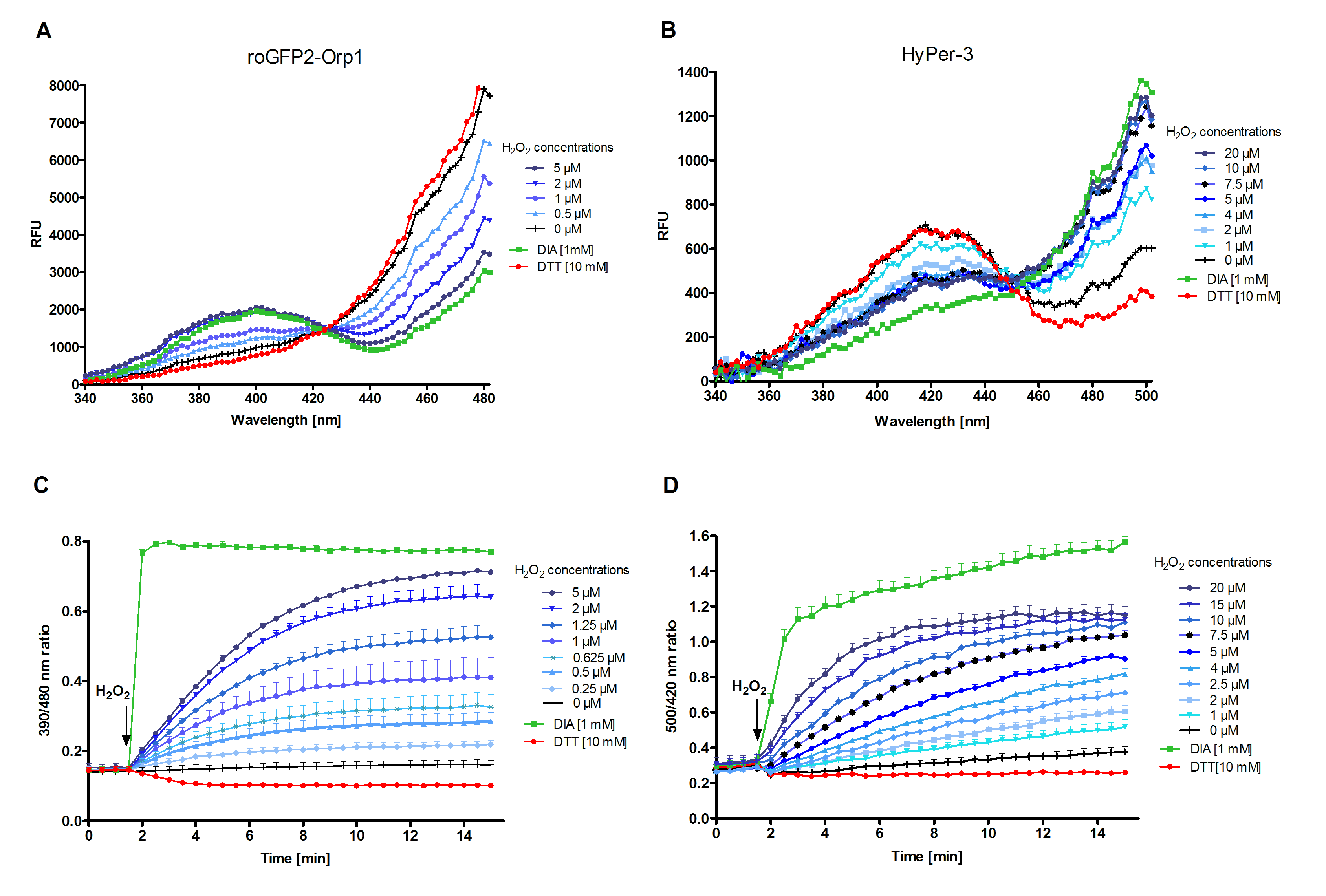

Supplement: S1 Fig — 5 μM of roGFP2-Orp1 (A) and HyPer-3 (B) were exposed to 1 mM DIA, 10 mM DTT, and different H2O2 concentrations in a microplate reader, which caused concentration-dependent changes in the excitation spectra. The recombinant proteins roGFP2-Orp1 and HyPer-3 have two excitation maxima at 390 nm and 480 nm (emission at 510 nm) (A), and 420 nm and 500 nm (emission at 530 nm) (B), respectively. Notably, roGFP2-Orp1 was fully oxidized by 5 μM H2O2 (A), whereas 20 μM H2O2 had to be applied to fully oxidize HyPer-3 (B). Furthermore, after 1.5 min baseline monitoring, recombinant roGFP2-Orp1 (C) and HyPer-3 (D) were treated with different concentrations of H2O2, 1 mM DIA, or 10 mM DTT and ratio changes were monitored for 15 min. For each concentration, data from three independent experiments were analyzed per data point. Mean and standard error of the mean (SEM) are shown. (TIF) [file pone.0174837.s001.tif]

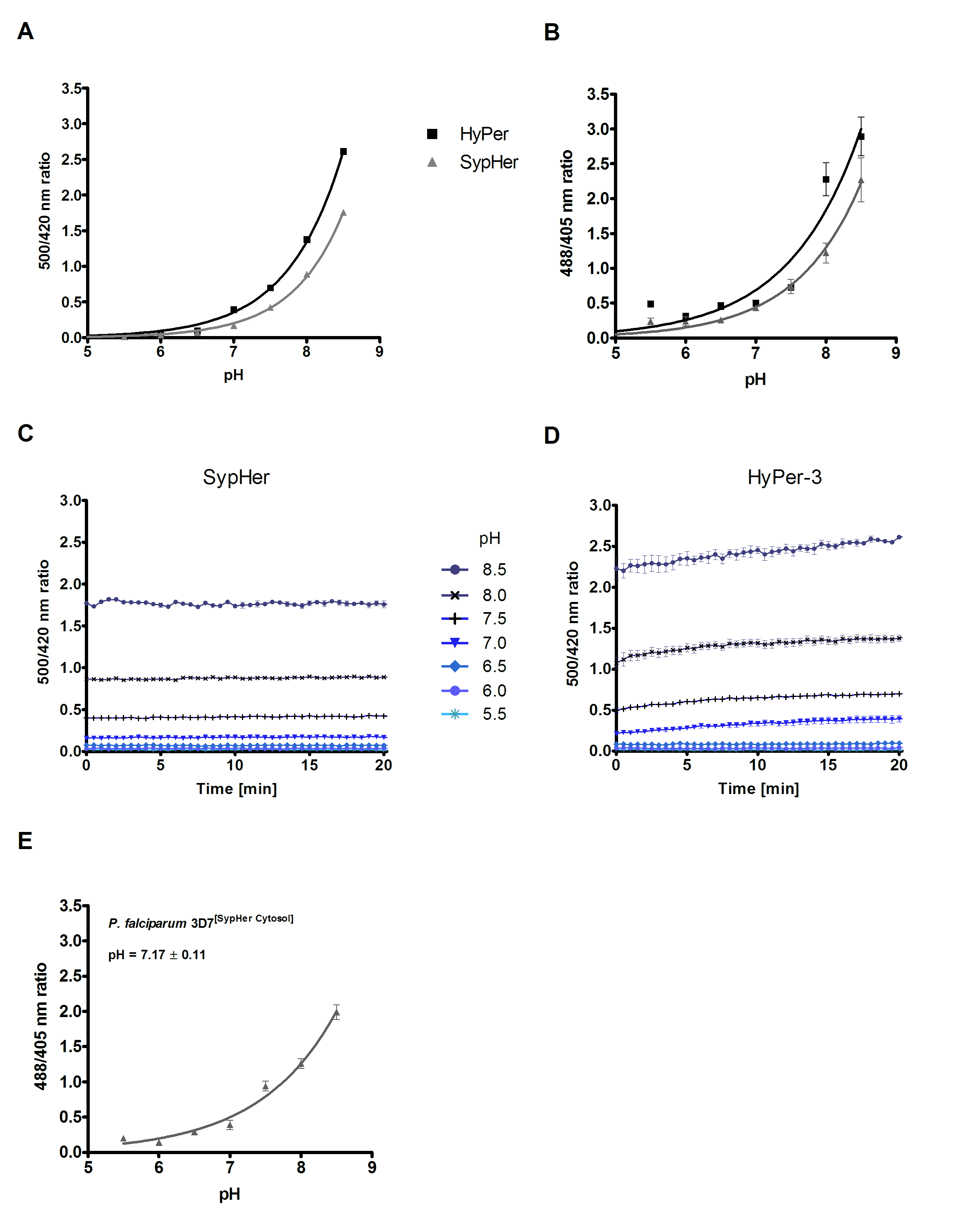

Supplement: S2 Fig — (A) Recombinant SypHer and HyPer-3 were suspended in pH buffers ranging from 5.5 to 8.5. Relative fluorescence units (RFUs) were measured at the emission wavelength of 530 nm and the 500/420 nm ratio was recorded. (B) In parallel, P. falciparum 3D7[SypHer]-transfected and 3D7[HyPer-3]-transfected parasites were studied at different pH values and the 488/405 ratio was recorded. In both in vitro (A) and in-cell (B) experiments, a different pH sensitivity of SypHer and HyPer-3 became evident. Only between pH 7–7.5 was the pH sensitivity of SypHer and HyPer-3 in the parasites comparable (B). Panels (C) and (D) show a time course for both sensors at different given pH values in vitro. Data indicate a higher stability of the SypHer probe. (E) Via calibration curves and nonlinear regression using the pH sensor SypHer, the cytosolic pH of P. falciparum 3D7 was determined to be 7.17 ± 0.11. As indicated, an exponential relationship exists between the 488/405 ratio of the sensor and the pH. For each pH value in in vitro measurements with the microplate reader, data from three independent experiments were included per data point. In living parasites, each data point represents ≥ 24 trophozoites analyzed in three independent experiments (CLSM detection). Mean and standard error of the mean (SEM) are shown. (TIF) [file pone.0174837.s002.tif]

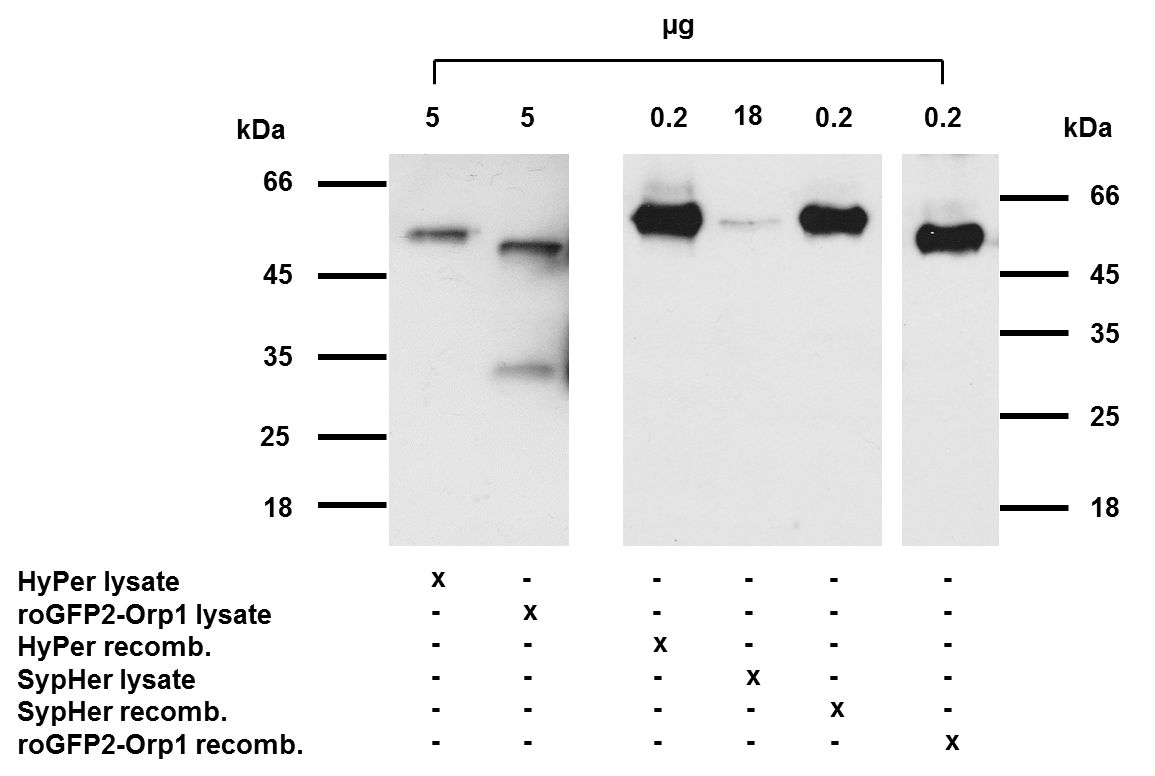

Supplement: S3 Fig — 0.2 μg of each recombinant purified protein was loaded, as well as 5 μg of parasite lysate expressing HyPer-3 and roGFP2-Orp1, respectively. For SypHer parasite lysate, 18 μg had to be applied because of its low transfection rate. Full-length roGFP2-Orp1 (49 kDa), HyPer-3 (52 kDa), and SypHer (52 kDa) probes are highlighted. For roGFP2-Orp1, a second band of ~ 27 kDa was also detected. (TIF) [file pone.0174837.s003.tif]

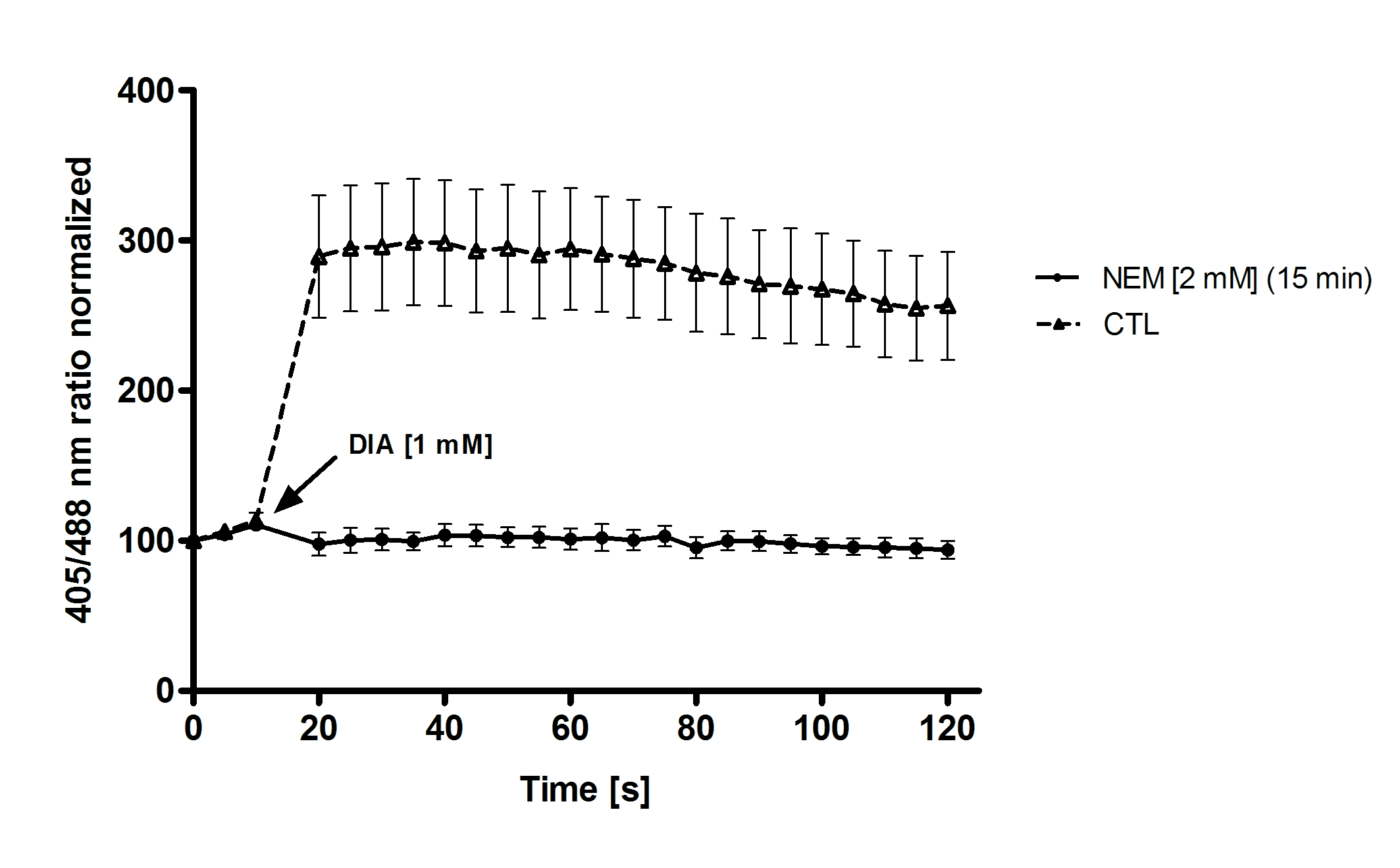

Supplement: S4 Fig — P. falciparum 3D7[roGFP2-Orp1] trophozoites were magnetically enriched and maintained at standard cell culture conditions for 2 h to recover. Parasites were then distributed to LoBind tubes (1 x 10^6^ iRBCs/100 μl) and incubated with 100 μM, 500 μM, 1 mM, 2 mM, 5 mM, 10 mM, 15 mM, and 20 mM NEM for 15 min to block the cysteine SH-groups. Subsequently, the parasites were exposed to 1 mM DIA to test blocking of the different NEM concentrations. The 405/488 nm ratio was measured via CLSM. Only the data of the 2 mM NEM incubation are shown, which was the lowest concentration showing full protective effects. (TIF) [file pone.0174837.s004.tif]
