# Supplementary material for: H2O2 dynamics in the malaria parasite Plasmodium falciparum
Source: PLoS One. 2017 Apr 3;12(4):e0174837. doi: 10.1371/journal.pone.0174837 (PMC5378400; doi:10.1371/journal.pone.0174837)
Supplement: S1 Table — (PDF) [file pone.0174837.s005.pdf]

S1 Table. Effects of antimalarial drugs on the redox ratio of recombinant roGFP2-Orp1 *in vitro.*

Drugs	Increase in fluorescence ratio [a]				Fold change of fluorescence ratio [b]			
[1 mM]	0 min	5 min	4 h	24 h	0 min	5 min	4 h	24 h
**ART**	No effect	No effect	0.24 → 0.39	0.44 → 0.56	–	–	1.63	1.27
**ATM**	No effect	No effect	No effect	No effect	–	–	–	–
**ATS**	No effect	No effect	No effect	No effect	–	–	–	–
**CQ**	No effect	No effect	No effect	No effect	–	–	–	–
**MQ**	No effect	No effect	No effect	No effect	–	–	–	–
**QN**	No effect	No effect	No effect	No effect	–	–	–	–
**CEA**	0.16 → 0.54	0.17 → 0.71	0.24 → 0.62	0.44 → 0.60	3.38	4.18	2.58	1.36
**FEA**	0.16 → 0.64	0.17 → 0.84	0.24 → 0.61	0.44 → 0.55	4.00	4.94	2.54	1.25
**MB**	0.16 → 0.73	0.17 → 1.07	0.24 → 1.03	0.44 → 0.95	4.56	6.29	4.29	2.16

[a] In this column the absolute change in the fluorescence ratio 390/480 nm of isolated recombinant roGFP2-Orp1 after incubation with the antimalarial compounds at 1 mM and different time points is shown. Furthermore, the basal ratio 390/480 nm of recombinant roGFP2-Orp1, which served as the starting point for the experiments, is given.

[b] In this column the fold change in the fluorescence ratio 390/480 nm of isolated recombinant roGFP2-Orp1 after incubation with the compounds at given concentrations and time points is shown.
