# Supplementary material for: H2O2 dynamics in the malaria parasite Plasmodium falciparum
Source: PLoS One. 2017 Apr 3;12(4):e0174837. doi: 10.1371/journal.pone.0174837 (PMC5378400; doi:10.1371/journal.pone.0174837)
Supplement: S2 Table — (PDF) [file pone.0174837.s006.pdf]

S2 Table. Effects of antimalarial drugs on the redox ratio of recombinant HyPer-3 *in vitro.*

Drugs	Increase in fluorescence ratio [a]				Fold change of fluorescence ratio [b]			
[1 mM]	0 min	5 min	4 h	24 h	0 min	5 min	4 h	24 h
**ART**	No effect	No effect	No effect	No effect	–	–	–	–
**ATM**	No effect	No effect	No effect	No effect	–	–	–	–
**ATS**	No effect	No effect	No effect	No effect	–	–	–	–
**CQ**	No effect	No effect	No effect	0.71→ 0.95	–	–	–	1.34
**MQ**	No effect	No effect	No effect	No effect	–	–	–	–
**QN**	No effect	No effect	No effect	No effect	–	–	–	–
**CEA**	0.31 → 0.47	0.34 → 0.72	0.46 → 1.13	0.71 → 0.89	1.52	2.12	2.46	1.25
**FEA**	0.31 → 0.58	0.34 → 1.02	0.46 → 0.93	0.71 → 0.93	1.87	3.00	2.02	1.31
**MB**	0.31 → 0.44	0.34 → 0.56	0.46 → 0.50	0.71 → 1.14	1.42	1.65	1.08	1.61

[a] In this column the absolute change in the fluorescence ratio 500/420 nm of isolated recombinant HyPer-3 after incubation with the antimalarial compounds at 1 mM and different time points is shown. Furthermore, the basal ratio 500/420 nm of recombinant HyPer-3, which served as starting point for the experiments, is given.

[b] In this column the fold change in the fluorescence ratio 500/420 nm of isolated recombinant HyPer-3 after incubation with the compounds at given concentrations and time points is shown.
