# Supplementary material for: H2O2 dynamics in the malaria parasite Plasmodium falciparum
Source: PLoS One. 2017 Apr 3;12(4):e0174837. doi: 10.1371/journal.pone.0174837 (PMC5378400; doi:10.1371/journal.pone.0174837)
Supplement: S3 Table — (PDF) [file pone.0174837.s007.pdf]

S3 Table. EC_{50} values of antimalarial drugs on *P. falciparum* 3D7 determined by the $[H]^3$-hypoxanthine incorporation assay described in [77].

Antimalarial drugs/redox-active compounds (abbreviations)	EC_{50} [nM]
Artemisinin (ART)	17.3
Artemether (ATM)	5.9
Artesunate (ATS)	4.4
Chloroquine (CQ)	8.6
Mefloquine (MQ)	8.0
Quinine (QN)	210
Coruleoellagic acid (CEA)	42
Flavellagic acid (FEA)	94
Methylene blue (MB)	3.3
