# Supplementary material for: H2O2 dynamics in the malaria parasite Plasmodium falciparum
Source: PLoS One. 2017 Apr 3;12(4):e0174837. doi: 10.1371/journal.pone.0174837 (PMC5378400; doi:10.1371/journal.pone.0174837)
Supplement: S4 Table — (PDF) [file pone.0174837.s008.pdf]

S4 Table. Primer sequences for cloning roGFP2-Orp1, HyPer-3, and SypHer into the expression vector pARL1a+ for in cell experiments, and for cloning HyPer-3 and SypHer into the expression vector pET28a+ for *in vitro* experiments.

**roGFP2-Orp1**	pARL1a+
OroGFP2Kpns	5’-atatGGTACCATGAGCAAGGGCGAGGAGC-3’
OOrp1Kpnas	5’-atatGGTACCTTATTCCACCTCTTTCAAAAGTTC-
**HyPer-3/SypHer**	pARL1a+
OHyPKpns	5’-atatGGTACCATGGAGATGGCAAGCCAGC-3’
OHyPKpnas	5’-atatGGTACCTTAAACCGCCTGTTTTAAAACTT-3’
**HyPer-3/SypHer**	pET28a+
OHyPNcoIs	5’-atatCCATGGAGATGGCAAGCCAGCAGG-3’
OpetHyPXho	5’-atatCTCGAGAACCGCCTGTTTTAAAACTTTA-3’

The restriction sites are underlined.
